# Efficiency, Profitability and Carbon Footprint of Different Management Programs under No-Till to Control Herbicide Resistant *Papaver rhoeas*

**DOI:** 10.3390/plants9040433

**Published:** 2020-04-01

**Authors:** Jordi Recasens, Aritz Royo-Esnal, Francisco Valencia-Gredilla, Joel Torra

**Affiliations:** Departament d’Hortofructicultura, Botànica i Jardineria, Agrotecnio, Universitat de Lleida, Alcalde Rovira Roure 191, 25198 Lleida, Spain; aritz.royo@udl.cat (A.R.-E.); francisco.valencia@udl.cat (F.V.-G.); joel.torra@udl.cat (J.T.)

**Keywords:** integrated weed management, winter cereal, poppy, environmental impact, economic cost

## Abstract

The present work examines the effects of different integrated weed management (IWM) programs on multiple herbicide-resistant *Papaver rhoeas* populations in terms of effectiveness, profitability and carbon footprint. With this aim a trial was established in a winter cereal field under no-till in North-Eastern Spain during three consecutive seasons. Four IWM programs with different intensification levels, from less (crop rotation, mechanical control, and no herbicides) to more intense (wheat monoculture with high chemical inputs), were established. The different strategies integrated in the four programs were efficient in managing the weed after three years, with increased effectiveness after management program intensification. Whereas low input program (which includes fallow season) represented less economic cost than the other programs, on average, no differences were observed on carbon foot print, considered as kg CO_2_eq kg^−1^ product, between the different programs, except in the crop rotation program due to the low pea yield obtained. The results from this study show that in the search for a balance between crop profitability and reduction of the carbon footprint while controlling an herbicide resistant population is challenging, and particularly under no-till. In this scenario the short term priority should be to reduce the presence of multiple herbicide resistant biotypes integrating the different available chemical, cultural, and physical strategies.

## 1. Introduction

In winter cereals, the adoption of integrated weed management (IWM) programs has been proven to be the best method for reducing weed infestation levels, compared with traditional cereal monocrop systems. Integrated weed management programs include different cultural (i.e., crop rotation, sowing delay, crop density, sowing pattern), physical (soil tillage, harrowing), and chemical strategies [1,2,3]. However, in Mediterranean semiarid areas of Spain, limited options are available for crop rotations, and crop sowing date is dependent upon autumn rainfall, which is often irregular and erratic. This scenario narrows the benefits available after crop harvest, thereby forcing most growers to adopt conservation tillage systems. Doing so implies savings in terms of time and economic inputs [4,5].

The most frequently employed conservation tillage system is direct drilling, which does not disturb the soil, apart from a few centimeters (cm) at sowing. In areas with conservation tillage in Spain, corn poppy (*Papaver rhoeas* L.) has become one of the most widespread broadleaf weed species [6]. Several studies have demonstrated that *P. rhoeas* is better adapted to no-till rather than to conventionally tilled cropping systems [7,8], based on the greater availability of seeds remaining in the 0- to 5-cm soil profile [9] and on the ability of small-sized seed species (≈1mm) to emerge from this environment. Moreover, the ability of *P. rhoeas* to infest and persist in arable fields can be attributed to the formation of a persistent seedbank, with an extended germination period and high fecundity rate [10]. 

In recent decades, *P. rhoeas* has become an increasing problem due to the appearance of biotypes resistant to synthetic auxin and/or acetolactate synthase (ALS)-inhibiting herbicides [11]. This problem was first observed in fields under conventional tillage, by which the implementation of different IWM programs demonstrated its effectiveness [6,12,13,14]. All these programs included crop rotations, alternative herbicides, sowing delay, mechanical control or fallow management. In no-till, where there is a greater reliance on herbicides, applying glyphosate before sowing is mandatory, and the use of different post-emergence herbicides creates a favorable scenario for the appearance of multiple resistance. A unique study focusing on the long-term effects on herbicide resistant *P. rhoeas* populations [15] different IWM programs (including some of the above mentioned strategies) better reduced the weed size population size under no-till (≈95%), compared to intensive tillage (≈86%). Therefore, crop rotation, sowing delay, or variations in the herbicide application timing under no-till are highly effective in mitigating the evolution of resistance.

On the other hand, Gan et al. [16] confirmed that the adoption of more intensified cropping systems (reducing the frequency of summer fallow, using high inorganic fertilizer and chemical inputs) increase crop yields compared to the traditional fallow-wheat or wheat monoculture. However, the increased use of inorganic fertilizers and pesticides in high-yielding systems contribute to the greenhouse gas emissions. These same authors highlighted that the integration of various cultural practices can substantially reduce herbicide inputs in crop production, and thus the carbon footprint. For example, the use of more competitive cultivars, higher sowing densities, and cereal–oilseed rape crop rotation, coupled with half rate (1/2x) of herbicides, achieved weed control levels similar to low-management programs (the use of less competitive cultivars, average sowing rates, and monoculture), coupled with a full herbicide application rate. Even though herbicides are small contributors to carbon footprint estimation [17,18], optimizing crop health with better agronomic management offers new opportunities for crop productivity improvement while reducing carbon footprints. In this sense, the adoption of conservation tillage is a recommended practice for enhancing carbon sequestration [19]. Unfortunately, the presence of herbicide-resistant weed populations in cereal crops offers a difficult context in which the application of low herbicide rates is discouraged, and their use with different sites of action (SoA) is recommended [20]. Therefore, the implementation of an IWM program for herbicide-resistant populations that will combine cultural and mechanical strategies with specific herbicide programs offers a scenario with a unidirectional challenge: To optimize the efficiency of the implemented strategies, while integrating cultural practices and the appropriate fertilizer and pesticide levels that will reduce the footprint of the crop products.

To date, there are no studies focusing on the long-term effects of various IWM intensification levels on herbicide-resistant *P. rhoeas* populations, which also consider the carbon footprint in the production system. Thus, the objective of this work was to study four three-year IWM programs in no-till, with different levels of intensification, for the control of a multiple herbicide-resistant *P. rhoeas* population, and analyze which of farming practices establish a better balance between grain production and carbon footprint.

## 2. Results

### 2.1. Climatic Data

The three growing seasons (Table 1) differed in terms of temperature and precipitation: 2014–2015 was the warmest (11.4 °C) but also with the most contrast, with a difference of 19.1 °C between the coldest and warmest month. This contrast was lower in 2015–2016, at 15.3 °C, and the growing season was the coldest (10.7 °C), although it had the warmest winter. The 2016–2017 growing season was average compared to the other two seasons (mean 11.2 °C), but had the most contrast between the coolest and warmest months (20.8 °C). Late frosts occurred on 30 April 2017, which affected the cereal grain production, but more so in wheat than in barley due to the different structure of their grain wrap. With respect to precipitation, 2014–2015 was the driest season (299 mm), but differed most in the distribution of rainfall along the seasons, having the wettest autumn (151 mm, Sep–Nov) and the driest spring (44 mm, Mar–May). The 2015–2016 season was the wettest season (363 mm). While autumn rainfall was average (116 mm, Sep–Nov), this was followed by a dry period for the next two months (9 mm, Dec–Jan), and a wet spring (181 mm). Finally, the 2016–2017 season was average with respect to the other two seasons (348 mm) with both autumn (125 mm) and spring (155 mm) being wet.

### 2.2. Density Changes

Densities were homogeneous and similar between different management plots at the beginning of the first season (2014–2015). Initial densities of *P. rhoeas* averaged 161 plants m^−2^ (Table 2). In this first season, three management programs were used (TRAD, CER, and CR), in addition to herbicide treatments and also a cultural management program (LI) was included. All four management programs significantly reduced (>99%) *P. rhoeas* densities by the end of the season, irrespective of the crop sowing date.

The initial density in the second season (2015–2016) was significantly lower (81 plants m^−2^ on average) than in the preceding season (Table 2), and ranged from 65 plants m^−2^ for CR to 96 plants m^−2^ for CER, however, the density for LI was higher (181 plants m^−2^). During this season, the first emergences of weeds were observed in mid-December, according with the low autumn rainfall (Table 1). For this reason, the TRAD sowing date had to be delayed and therefore differences with the two delayed sowing managements (CER and CR) were reduced to a few days. At the end of the season, densities were significantly reduced (>99%) for all management programs assessed, with values ranging between 0 and 0.63 plants m^−2^.

The analysis of the initial *P. rhoeas* density in the third season (2016–2017) revealed significant differences between management programs (Table 2). LI resulted in highest weed densities (156 plants m^−2^), followed by CR, at 69 plants m^−2^. The other two programs (TRAD and CER) lowered weed densities at the start of the season: 4 and 11 plants m^−2^, respectively. The density observed in LI when the crop was sown on 2 December (156 plants m^−2^) was lower than densities registered one month before (4 November) (730 plants m^−2^, data not shown), when barley was sown under the other three programs. Despite the initial differences, densities were significantly reduced by all managements: From 80% by TRAD, and up to 99% in LI.

The initial density estimated in December 2017 ranged, without significant differences, from 16 plants m^−2^ to 34 plants m^−2^ for TRAD, CER, and CR programs (Table 2), which was a density reduction of 93%, 82%, and 76%, respectively. Nevertheless, the initial density in LI was significantly higher (80 plants m^−2^) that the other three programs, thereby making it the management associated with the lowest density reduction (35%) after three seasons.

### 2.3. Crop Yields

Yields differed between the strategies as a consequence of crop and sowing date variation, in addition to the climatic conditions each season (Table 3). The cereal yield in the first season (2014–2015) varied between 2923 kg ha^−1^ for barley (TRAD) and 2683 kg ha^-1^ for wheat (CER) when sowing was performed in October, while a sowing delay to 13 December severely decreased barley yield (989 kg ha^−1^, LI). Conversely, a wet spring allowed for higher cereal yields in the second season (2015–2016) (4343 kg ha^−1^ in CR; 6723 kg ha^−1^ in CER and 6783 kg ha^−1^ in TRAD). Contrary to the first season, a sowing delay to 2 December (LI) in 2016–2017 permitted higher barley yields (3891 kg ha^−1^) than when it was sown on 4 November (2631 kg ha^−1^, TRAD) or than wheat (2919 kg ha^−1^ and 3514 kg ha^−1^ for CER and CR, respectively). Even still, differences were not significant, which could be explained by the late frost registered at the end of April 2016, which adversely affected the cereal in an advanced phenological stage.

The pea yield in 2014–2015 was very low (183 kg ha^−1^, CR). The economic income that the pea yields represent over all three seasons differed significantly between management programs. TRAD and CER programs averaged 2000 € ha^−1^; whereas CR averaged 1284 € ha^−1^, due to the low income generated by pea yield the first season. Management with LI generated only 792 € ha^−1^ since a fallow was established the second season.

### 2.4. Economic Cost of Different Programs

The estimated cost for the agronomic tasks carried out each season in each program also differed (Table 4). The total expenses were slightly higher in CER and CR (1609 € and 1694 €, respectively) than in TRAD (1539 €). The total cost of LI was the lowest (1119 €) based on the fallow established during the 2015–2016 season.

### 2.5. Carbon Footprint

Table 5 shows the carbon footprint, in both kg CO_2_eq kg^−1^ product and kg CO_2_eq ha^−1^, of the different management techniques across the three growing seasons. Regarding this input per kg of product, a higher value was obtained in CR the first season (15.2 kg CO_2_) due to the lower pea yield. For the other managements, this value is always lower than 1.2 kg CO_2_ except for LI in the first season (2.8 kg CO_2_). When footprint is estimated per ha, a similar input was obtained between programs across the three seasons (ranging between 2791 and 2837 kgCO_2_eq ha^−1^), except for LI (with 2016 kgCO_2_eq ha^−1^) according to the fallow establishment the second season.

The fabrication and application of fertilizers supposes the most important contribution to the carbon footprint of all management programs (Appendix A), with similar averages of 1633 kg CO_2_ ha^−1^ and 785 kg CO_2_ ha^−1^, respectively, for TRAD, CER and CR managements. In LI program, these values were lower, with 1244 kg CO_2_ ha^−1^ for fabrication and 456 kg CO_2_ ha^−1^ for application. Footprint average for diesel fieldwork ranged between 213 kg CO_2_ ha^−1^ (in TRAD and CER managements) and 174 kg CO_2_ ha^−1^ for LI, whereas agrochemicals fabrication had lower averaged values of 47, 50, 81, and 57 kg CO_2_ ha^−1^ for TRAD, CER, CR, and LI, respectively.

## 3. Discussion

### 3.1. Herbicide Resistance and Integrated Weed Management

The presence of a multiple herbicide-resistant corn poppy population was confirmed. In the greenhouse assessment of this population, no mortality was observed at the commercial label rates for 2,4-D and tribenuron. These results confirm the increasing presence of multiple herbicide-resistant biotypes in the region, not only in fields with conventional tillage, but also in those with no-till [15]. Due to the small size of *P. rhoeas* seeds, this species is capable of remaining in the soil top layers (0 to 5 cm), and can emerge from the most superficial soil profile [9]. Herbicides alone are usually not enough to control these multiple herbicide-resistant populations, and the adoption of IWM programs becomes essential [20].

### 3.2. Three-Year Assessment of Different Weed Management Programs

In this study, the established managements with more herbicide inputs (programs TRAD and CER) were the most efficient in reducing initial *P. rhoeas* densities from 2014 to 2017, which varied between 93% and 82% after three years, respectively. Managements with crop rotations and non-chemical strategies (programs CR and LI) resulted less effective (76% and 35%, respectively) (Table 2). On the other hand, the control of *P. rhoeas* achieved every season was always higher than 97%, except the last season in TRAD (81%). These data highlight the importance of the seed bank longevity in this species to persist in cereal fields [21] even with high control efficacies, there still were seeds in the soil. Moreover, few plants potentially surviving to the management strategies can be enough to replenish the seed bank due to its high fecundity [10]. In LI, fallow shredding was done in early June 2016 because most plants were just starting to bloom, but the high weed density (181 plants m^−2^) could mask plants already fructifying and that might finally contribute to the seed bank. Therefore, to control multiple resistant *P. rhoeas*, as many other herbicide resistant weed species, timing of the control method is key to achieve successful results [22].

On the other hand, after three seasons, initial densities in the third season (between 16 and 80 plants m^−2^), showed the irregular expression of the seed bank across time and managements. For the two more efficient programs (TRAD and CER), initial densities (16 and 29 plants m^−2^, respectively) were lower than those observed the preceding season or compared to the other programs CR and LI (34 and 80 plants m^−2^, respectively). It is known that under Mediterranean climate conditions, the extent and amount of corn poppy emergence is determined by temperature and rainfall [23], and growth, fecundity and dormancy of seeds differs among established emergence cohorts [10]. In this respect, the results obtained for the four programs highlights that establishing adequate strategies to minimize the seed bank replenishment, together with the annual control of the different emerged cohorts are essential for this species. Torra et al. [15] confirmed significant reduction of *P. rhoeas* densities after three years of management under no-till, where promotion of early emergence could result from higher soil water content under no-till, thereby making delayed sowing a more effective strategy. However, in our study, emergences differed between seasons. In autumns with enough humidity (2014–2015 and 2016–2017) the first emergences were detected in October, whereas a dry summer and autumn (2015–2016) delayed the emergence of *P. rhoeas* to mid-December.

Multiple herbicide resistant *P. rhoeas* populations have been controlled with PRE and/or POST herbicide treatments with alternative SoA, even with very high infestation levels [14,24]. The same has been found in fields subjected to no-till [15]. The same herbicides were also effectively used in our experiment (>97% of control), although TRAD failed to some extent in the third season (81%). Glyphosate applied during pre-seeding is a common practice for conservation agriculture in the area [15,25] as in this trial, which aided in controlling the *P. rhoeas* population. In any case, the different strategies integrated in the four programs herein were efficient in managing the weed in all cases across time, as long as appropriate actions are carried out, according to the biology (demography and phenology) of the weed population.

### 3.3. Yields and Economic Income

Gross income was variable between programs and seasons, and mostly dependent on crop type (price) and yields achieved (Table 3). The net income after three seasons was only profitable (above 0 €) for TRAD and CER programs when balancing gross incomes (Table 3) and costs (Table 4). The fallow year in LI or very low pea yields in CR explain low incomes in these managements. For this reason, establishment of less profitable crops or with more technical issues are not recommended to include in IWM programs. To optimize the management of herbicide-resistant weeds, introducing crop rotation should be the first cultural practice considered by farmers [26], and has even been demonstrated as profitable for the control of other surrounding weeds [1]. Crop prices and yields achieved every season will be among the main factors driving *P. rhoeas* management decisions by farmers, together with fuel, fertilizer and seed costs [13].

### 3.4. Reconciling Carbon Footprint, Efficiency, and Profitability

Across the three seasons, the average differences between programs on carbon footprint are directly related to the crop rotation or fallow employed. In all programs, fertilizers (fabrication and application) are the major contributor to footprint, with percentages close to 85% of the total kg CO_2_ ha^−1^ (Appendix A) These results are in accordance with those obtained by Gan et al. [27]. These authors proposed the inclusion of N-fixation crops to reduce the use of fertilizers by diversifying cropping systems with oilseed, pulse, and various cereal crops, compared with cereal-based monoculture systems. However, in Mediterranean semi-arid areas, limited options are available for crop rotations. In our case, the inclusion of pulse crops (such as pea in the CR program) and with delayed sowing, is not recommended as alternative to cereal monocrop (as TRAD and CER programs) due to the difficulties for the crop establishment in no-till, and for the risk of obtaining low yields in dry seasons. It is important to notice that this yield data for pea come from a single trial a single season and therefore it should be considered with caution. For this reason, CR program, on the three-seasons average, had the highest carbon footprint input per unit of product compared to the other programs. On the other hand, the inclusion of a fallow year (program LI) did not represent a higher contribution to the carbon footprint (kg CO_2_ kg^−1^ of product) compared to those more intensified programs (TRAD or CER). These results disagree with Liu et al. [18], who appointed that the more intensified wheat cropping practices deliver lower carbon footprint. These differences depend on the intervention made in fallow. In our case, LI was shredded, and the estimated footprint was assigned to the kg of grain of the others two seasons. Furthermore, the fabrication of agrochemicals (herbicides included) and the various fieldwork tasks (application of herbicides included) represent, jointly, between 9.3% (TRAD) and 11.4% (LI) of the total kg CO_2_ ha^−1^ of the program (Appendix A). These values are according with Lal [17] and Gan et al. [27], who found that the portion of footprint from pesticides used in agriculture is generally small.

Previous studies showed that is possible to reduce the carbon footprint with the adoption of improved farming techniques (i.e., crop rotation with legumes), thereby optimizing system performance [16,18]. However, those studies did not consider the profitability of the cropping system by analysing the balance between economic inputs and costs. The results from our study show that in the search for a balance between crop profitability and minimizing the carbon footprint while controlling an herbicide resistant population is challenging, and particularly under no-till. In this scenario the short term priority should be to reduce the presence of multiple herbicide resistant biotypes, integrating the different available chemical, cultural and physical strategies. Moreover, in a Mediterranean climate where yields can substantially change due to variable precipitations, the implementation of IWM programs will become more difficult. Volatility in annual crop prices and seed costs represents an additional handicap to implementing more sustainable and integrated crop production systems. Henceforth, it is crucial that farmers and policy makers align their goals of maintaining profitability while reducing carbon footprint and environmental impacts, which can only be done if governments promote farmers’ behavior with proper subsidy policies [13].

## 4. Material and Methods

### 4.1. Site Description

A field trial was established during three consecutive growing seasons (2014–2015, 2015–2016, and 2016–2017) in a commercial winter cereal field in Foradada (41°52′15″ N; 01°01′18″ E, 269 m.o.s.l.), Lleida, North-Eastern Spain. The field presented a high infestation of *P. rhoeas* and had been under production with no-till winter cereals for 10 seasons prior. During the previous season (2013–14), high infestation levels had forced the shredding of the crop in May, before seed maturity and seed rain of *P. rhoeas*. In 2011–12 and 2012–13, both PRE (pendimethalin) and selective POST (2,4-D plus florasulam) herbicides were used for the control of the weed. The field had a 3% slope to the North, and the soil structure was 42% sand, 20% clay and 38% silt, with 3.25% organic matter and a pH of 7.9.

### 4.2. Characterization of the Herbicide Resistance

Seeds from the experimental field were collected and stored during summer 2015 as a potential multiple herbicide-resistant (HR) population. In autumn, an experiment was conducted with this population and with one susceptible (SC) population from Almenar, 40 km away from the experimental site. Seeds were sterilized in a 30% hypochlorite solution and sown in petri dishes containing moistened filter paper. Petri dishes were placed in a growth chamber at 20/10 °C day/night and 16-h photoperiod under 350 µmol photosynthetic photon-flux density m^−2^ s^−1^. After 14 d, seedlings were transplanted to 3 × 3 × 4 cm plastic filled with a mixture of silty loam soil 40% (*w*/*v*), sand 30% (*w*/*v*), and peat 30% (*w*/*v*). Five seedlings were transplanted per pot, and were later thinned to four per pot. When both populations (HR and SC) achieved the 5- to 6-leaf stage (5–6 cm), 2,4-D herbicide (2,4-D ethyl-hexyl 600 g a.i. L^−1^, EC) (Esteron 60; DOW Agrosciences) was applied at 0, 600 (1x the field rate), 2400, and 3200 g a.i. ha^−1^, and the ALS inhibitor tribenuron (tribenuron-methyl 500 g a.i. kg^−1^, WSG) (Granstar 50 SX; DuPont) was applied at 0, 18.7 (1x), 75, and 300 g a.i. ha^−1^. Four replicates (pots) were included for each population and dose. Herbicides were applied using a precision bench sprayer delivering 200 L ha^−1^ at a pressure of 215 kPa. Pots were placed in a greenhouse at the University of Lleida, Spain, and watered regularly. Four weeks after treatment, plants from each dose were harvested (above ground). Samples were dried at 65 °C for 48 h, and dry weights were measured. For the HR population, the results obtained on percentage of reduction of dry weight, respect to control were 0%, 34% and 87%, at doses 1x, 4x and 8x, respectively, for 2,4-D; and 0%, 21%, and 43% for doses of 1x, 4x and 16x, respectively, for tribenuron. On the contrary, the percentages of reduction in dry weight for the SC population were 100% at all doses and for both herbicides, thereby confirming the presence of multiple resistant biotypes in the HR population from Foradada.

### 4.3. Integrated Weed Management Assessments

Each management program integrated different strategies, such as crop rotation, delayed sowing date, crop density, fallow, and herbicide rotation, resulting in four management programs of decreasing agronomical input degree (Table 6): (1) traditional (TRAD), barley was sown the three seasons and the control of weeds was by herbicides; (2) cereal rotation (CER), wheat (*Triticum aestivum* L.) was sown the first and third season and barley (*Hordeum vulgare* L.) was sown delayed the second season, and the control of weeds was by herbicides; (3) crop rotation (CR), pea-barley-wheat, delaying barley sowing the second season and changing, each season, the herbicides according to different site of action (SoA); (4) low input (LI), barley was sown delayed and at high density the first and third seasons, field was felt in fallow the second season and weeds were controlled mechanically by flexible rod harrow in cereal and by shredding in fallow. The experimental design was a complete randomized block with four replicates. Each management plot measured 15 m × 8 m (120 m^2^) to facilitate sowing, herbicide application and harvest, and was surrounded by a 10 m alley to buffer the trial. Field management activities were performed with a tractor John Deere 6910 with 140 CV, incorporating, when necessary, equipment for sowing, herbicide application, fertilization or shredding, according to its use.

For the 2014–2015 season, sowing was performed on 31 October in TRAD, 24 October in CER, 23 January in CR and 13 December in LI; in season 2015–2016, sowing was performed on 6 November in TRAD and on 12 November in CER and CR; and in season 2016–2017, sowing was performed on 4 November on TRAD, CER and CR and on 2 November on LI (Table 6). Sowing was done with a 4 m wide no-till disc drill.

In all management programs, glyphosate was applied every season prior to the crop sowing. To prepare for the second season, plots were shredded in September to remove *Kochia scoparia* (L.) Schrad. and other summer weeds. Herbicides were applied with a backpack plot sprayer using a 2-m wide boom, calibrated to deliver 300 L ha^−1^ of water at 253 kPa pressure. All details regarding herbicide applications and mechanical control are summarized in Table 7 and Table 8. For each season, fertilizer was applied before sowing at 70 UPN and then again at 100 UPN in February.

### 4.4. Data Collection

Climatic data were obtained from the automatic meteorological station in Oliola, (XAC public weather mesonet, www.ruralcat.net), located 15 km from the experimental field.

*P. rhoeas* density was quantified monthly, from sowing to harvest, by randomly throwing ten 0.10 m^2^ frames into each plot. Depending on the crop sowing date of each program, initial densities were estimated between December and February each season. The three-year experiment officially ended in June 2017, though *P. rhoeas* densities were also estimated at the beginning of the 2017–2018 season, in December 2017. All field plots were harvested on 22 July 2015, 12 July 2016 and 3 July 2017 with a micro-harvester (Wintersteiger classic plot combine micro-harvester).

Estimation of the annual income garnered for each crop was calculated according to the standard crop prices used by the agricultural cooperative of Agramunt: June 2015: barley, 178.5 € t^−1^; wheat, 192.5 € t^−1^; field pea, 240 € t^−1^; June 2016: barley, 152 € t^−1^; June 2017: barley, 158 € t^−1^; wheat, 165 € t^−1^.

For each management program, the literal costs of the treatment(s) and agronomic tasks were estimated using data from the Department of Agriculture of the Catalan Government (Generalitat de Catalunya), and autoestimations elaborated by farmers’ associations from the Regional Office of Urgell (Oficina Comarcal de l’Urgell, Lleida). Only specific tasks carried out in the field were considered and the cost of pesticides and fertilizers were estimated according to the public price for the three surveyed seasons. Data from equipment ownership, maintenance and reparation costs and field rental were considered the same for all programs and are not included in the analysis.

### 4.5. Estimation of Carbon Footprint

The carbon footprint of a product is a measure of greenhouse gas (GHG) emissions and an indicator used to assess the impact on global warming (GW). In this sense, the carbon footprint was estimated according to the model proposed by IPCC [28] and the result is expressed in units of equivalent kg CO_2_ kg product^−1^. The estimation of a product’s carbon footprint is based on the Life Cycle Analysis (LCA), following the general guidelines established by ISO 14040 and 14044, and concreted for carbon footprint in the ISO 14067, GHG Protocol [29] and PAS 2050:2011 [30]; the latter with a special version PAS 2050-1:2012 (BSI, 2012) for horticultural products.

In all cases, the spatial limits of the system were considered to be from the origin of raw materials and other energy sources used throughout the cropping season, to the gate where the product (grain) is transported and stored. On a temporal scale, the system is the cropping season, counted from the time of harvest of the previous season to the current one. The calculations are made over the surface unit of the field plot (hectare), considering the whole unit homogeneously. The production and consumption of supplies and the residues generated are referenced by hectare (i.e., kg of product ha^−1^).

The functional unit used in the LCA is 1 kg of farm product obtained per unit of surface. In the inventory, production and consumption data are referenced to the hectare as surface unit (i.e., kg product ha^−1^). In the final calculation, equivalent CO_2_ emissions (kg CO_2-eq_) are expressed as kg of product (kg CO_2-eq_ kg^−1^ product).

The basic formula (Equation (1)) to estimate the carbon footprint of a product is:

(1) $$HC=\sum_{i}HC_{i}=\sum_{i}\left(FC_{i}\cdot VA_{i}\right)$$

where *HC_i_* is the carbon footprint for the activity or process vector *i*; *FC_i_* is the conversion factor for the activity or process vector *i* obtained from the dataset Ecoinvent v3 [31] using the methodology IPCC [28] and the category “climate change GWP 100_a_” in the units kg CO_2eq_ activity unit^−1^; and *VA_i_* is the variable of activity for the vector process *i* (activity unit per functional unit).

The vectors are the following:

(1) Diesel oil: fabrication and combustion of diesel oil (CO_2_ emissions derived from the diesel combustion by farm operations).
(2) Synthetic and organic fertilizers: fabrication of synthetic fertilizers (N-P-K) plus gas emissions of N_2_O (direct and indirect emissions deriving from the application of both fertilizer types to the soil).
(3) Plant protection products: fabrication of fungicides, insecticides and herbicides.
(4) Transport of the products and raw materials: transport of farm products from the farm-gate to the store and of raw materials (fertilizers and plant protection products) from the distribution centre to the farm-gate.
(5) Transport and residues management: transport materials (wood or plastic boxes), and packaging plant protection products, are considered residues. Other plastic, wood or iron packaging materials are not included. It is assumed that the transport to the recycling centre or to the dump is made by the farmer himself.
(6) Infrastructure use: the redemption of different materials used for infrastructure (i.e., steel or rubber from fieldwork-related machinery). In these cases, the gasses emitted during the construction or operation of infrastructures should be amortized during the lifespan assigned to each infrastructure.

### 4.6. Vectors and Conversion Factors

To estimate the carbon footprint of different treatments and of the work involved with field conversion, factors of kilogram or liter consumed to kgCO_2_-eq were used. The vectors used and their respective conversion factors (Table 9) were obtained from the Ecoinvent 3.0 database (ECOINVENT, 2019). The distribution model of the environmental charge from the relevant Ecoinvent 3.0 database (Allocation Model) is “Allocation, ecoinvent default”. It was crucial to assess the impact of the use of diesel oil in fieldwork, and to add the emissions derived from gas combustion as a consequence of fuel use. To practical effects, the value is obtained multiplying the amount of diesel oil reported by functional unity by the factor 3.16 kg CO_2_-eq kg^−1^ diesel (obtained from “Elementary Exchanges Data” for the tillage activities) in the Ecoinvent v3 database, which included CO_2_, N_2_O, CO, and CH_4_ atmospheric emissions caused by diesel oil combustion. This value is added to the impact of diesel oil production.

### 4.7. Statistical Analysis

For the field experiment, the effect of different strategies on both initial and final *P. rhoeas* densities each season and over the changes (%) on initial density across the three complete seasons, were tested with linear mixed-effects models (LMM). The strategies were established as fixed factors and repetitions as random factors. *P. rhoeas* density data were transformed if needed (log (x + 1) or √ (x + 0.5)) prior to analysis to normalize data distribution. A post hoc Tukey’s pairwise comparison was employed to test differences between strategies (at *P* < 0.05). For the yield economic income, the analysis was performed for the overall combined income of the three seasons with a parametric one-way analysis of variance. The yields were not compared season by season since we wished to consider the three-year management as a whole. In a similar way was performed the analysis of carbon footprint of CO2eq kg^−1^ product. Analysis were performed with JMP Pro 14 software (SAS Institute 2010. SAS Campus Drive, Cary, NC 27513, USA. SAS Institute, Inc.).

## Figures and Tables

**Table 1:** Monthly temperature and precipitation during the three growing seasons.

Months	Mean Temperature (°C)			Precipitation (mm)		
	2014–2015	2015–2016	2016–2017	2014–2015	2015–2016	2016–2017
September	20.0	16.9	19.8	48.7	73.8	15.4
October	15.5	13.4	14.0	21.0	8.6	42.7
November	9.6	8.1	7.5	81.1	34.0	66.6
December	3.2	5.2	3.1	7.5	2.9	7.2
January	2.3	5.4	1.7	10.3	5.7	16.5
February	3.5	5.7	6.7	5.8	47.8	18.8
March	9.0	6.9	9.0	24.0	31.6	89.3
April	12.2	10.7	11.3	15.9	81.4	48.0
May	17.6	14.1	16.7	4.0	68.4	17.7
June	21.4	20.5	22.5	81.1	9.2	25.9
Mean(°C)/Total (mm)	11.4	10.7	11.2	299.4	363.4	348.1

(Oliola Meteorological station).

**Table 2:** Initial (ID) and final (FD) *Papaver rhoeas* density means (plants m^−2^) under different management programs during three consecutive seasons. DR: annual reduction in density, FD relative to ID in %. Last column represents the reduction of ID in December 2017.

Season	2014–2015			2015–2016			2016–2017			2017	Change (%) in ID (2014–17)
Program	ID	FD	DR (%)	ID	FD	DR (%)	ID	FD	DR (%)	ID	
1. TRAD	218 (a)	0.75 (ab)	99.5	83 (a)	0.03 (a)	99.9	4 (a)	0.78 (a)	80.5	16 (a)	93 (a)
2. CER	162 (a)	0 (b)	100	96 (a)	0.05 (a)	99.9	11 (a)	0.30 (a)	97.2	29 (a)	82 (a)
3. CR	142 (a)	0.78 (a)	99.2	65 (a)	0.63 (b)	99	69 (ab)	1.03 (a)	98.5	34 (a)	76 (a)
4. LI	122 (a)	0.28 (ab)	99.2	181 (a)	0 (a)	100	156 (b)	1.03 (a)	99.8	80 (b)	35 (b)

Values with different letter are significantly different according to the Tukey’s test (*p ≤ 0.05*).

**Table 3:** Yield (kg ha^−1^) and Gross income (€ ha^−1^) (+SE) obtained each season for each established management program. The result of the statistical analysis for the overall income after three years of each program is included in the last column.

Season	Yield			Gross Income *			
	2014–2015	2015–2016	2016–2017	2014–2015	2015–2016	2016–2017	Total
Program	kg ha^−1^	kg ha^−1^	kg ha^−1^	€ ha^−1^	€ ha^−1^	€ ha^−1^	€ ha^−1^
TRAD	2923 ± 541 (a)	6783 ± 753 (a)	2631 ± 682 (a)	522 ± 97	1031± 76	416 ± 108	1969 ± 142 (a)
CER	2683 ± 500 (a)	6723 ± 497 (a)	2919 ± 1004 (a)	517 ± 96	1022 ± 114	481 ± 166	2020 ± 292 (a)
CR	183 ± 31 (c)	4343 ± 463 (b)	3514 ± 704 (a)	44 ± 7	660 ± 70	580 ± 116	1284 ± 178 (b)
LI	989 ± 137 (b)	-	3891 ± 744 (a)	177 ± 25	-	615 ± 118	792 ± 122 (c)

Values with different letter are significantly different according to the Tukey’s test (*p ≤ 0.05*). * Crop Prices (€ t^−1^) found in the Materials and Methods section.

**Table 4:** Economic cost (€) of different agronomic tasks carried out in each management program during three seasons. See text for more details.

Season	Cost of Management Programs (€)			
	TRAD	CER	CR	LI
2014–2015	509.14	516.52	601.64	512.27
2015–2016	506.52	521.52	521.52	59.87
2016–2017	524.12	571.12	571.12	547.19
Total 3 seasons (€)	1539.78	1609.16	1694.28	1119.33

**Table 5:** Carbon footprint (kg CO_2_- eq kg product^−1^ and kg CO_2_- eq ha^−1^) for four different management programs established in a winter cereal field during three consecutive seasons.

Kg CO_2_ eq kg product^−1^	Season			Average ^1^	Kg CO_2_ eq ha^−1^	Season			Average
	2014–2015	2015–2016	2016–2017			2014–2015	2015–2016	2016–2017	
TRAD	0.9	0.4	1.2	0.8 (a)	TRAD	2725	2553	3096	2791
CER	1	0.4	1.1	0.8 (a)	CER	2710	2558	3115	2794
CR	15.2	0.6	0.9	5.6 (b)	CR	2774	2586	3149	2837
LI	2.8	n.a*	0.8	1.2 (a)	LI	2722	182	3146	2016

*n.a.: not applied. The carbon footprint generated the second season by shredder in fallow in LI, is assigned to the yield of the others two season. ^1^ Values with different letter are significantly different according to the Tukey’s test (*p* ≤ 0.05).

**Table 6:** Characteristics of each management program and season: TRAD, barley monocrop with post emergence application of herbicides; CER, wheat-barley rotation with pre and post emergence application of herbicides; CR, crop rotation (pea-barley-wheat) with chemical and cultural management; LI, low input non-chemical management. See text for more details. Var.: variety; Dens: crop density; DS: date of sowing. Var: variety; Dens: crop density; DS: Date of sowing.

Season 2014–2015					Season 2015–2016				Season 2016–2017			
Program	Crop	Var.	Dens. kg ha^−1^	DS	Crop	Var.	Dens. kg ha^−1^	DS	Crop	Var.	Dens. kg ha^−1^	DS
TRAD	Barley	Cometa	200	31/10	Barley	Meseta	200	6/11	Barley	Meseta	200	4/11
CER	Wheat	Verdun	200	24/10	Barley	Graphic	200	12/11	Wheat	Astur N	200	4/11
CR	Pea	Mystic	200	23/01	Barley	Graphic	200	12/11	Wheat	Artur N	200	4/11
LI	Barley	Cometa	220	13/12	Fallow	-	-	-	Barley	Graphic	220	2/12
Program	Crop	Herb	Cultural Management		Crop	Herb.	Cultural management		Crop	Herb.	Cultural management	
TRAD	Barley	Post ^1^	-		Barley	Post ^4^	-		Barley	Post ^7^	-	
CER	Wheat	Post ^2^	-		Barley	Post ^5^	SD		Wheat	Pre ^8^ + Post ^9^	-	
CR	Pea	Pre ^3^	-		Barley	Post ^6^	SD		Wheat	Pre ^10^ + Post ^1^	-	
LI	Barley	-	SD/HD/MC		Fallow	-	Shredding		Barley	-	SD/HD/MC	

Herb: herbicide; Pre: pre-emergence; Post: post-emergence SD: sowing delay; HD: high crop density; MC: mechanical control. Herbicides: 1: bromoxynil+MCPP; 2,4,5,6: aminopyralid+florasulam; 3: pendimethaline+imazamox; 7,9,11: chlortoluron+diflufenican; 8,10: isoxaben.

**Table 7:** Herbicides applied during the three growing seasons. SL soluble concentration; EC, emulsionable concentration; WG, water dispersable granulate; SC, concentrated suspension.

Product	Active Ingredient	Formulation	Dose
Touchdown	glyphosate (36%)	SL	2 L ha^−1^
Image Gold	bromoxynil + mecoprop	EC	1.75 L ha^−1^
Intensity	aminopyralid + florasulam	WG	33 g ha^−1^
Mutual	pendimethalin + imazamox	EC	4 L ha^−1^
Legacy plus	chlortoluron + diflufenican	SC	2 L ha^−1^
Rokenyl	Isoxaben	SC	0.2 L ha^−1^

**Table 8:** Dates of herbicides application and mechanical control in each management program.

Treatment	Management Program			
	1. TRAD	2. CER	3. CR	4. LI
Presowing glyphosate	20/10/2014	20/10/2014	12/01/2015	11/12/2014
Bromoxyil + MCPP	12/01/2015			
Aminopyralid + florasulam		12/01/2015		
Pendimenthalin + imazamox			26/01/2015	
Flexible rod harrow				18/03/2015
Shredding	15/09/2015	15/09/2015	15/09/2015	15/09/2015
Presowing glyphosate	06/10/2015	10/12/2015	10/12/2015	20/01/2016
Aminopyralid + florasulam	28/12/2015	19/02/2016	19/02/2016	
Shredding				04/06/2016
Presowing glyphosate	06/11/2016	06/11/2016	06/11/2016	01/12/2016
Isoxaben		07/11/2016	07/11/2016	
Diflufenican + chlortoluron	12/12/2016	12/12/2016	12/12/2016	
Flexible rod harrow				01/03/2016

**Table 9:** Vectors, vectors family and conversion factors used (Ecoinvent 3.0).

VECTOR FAMILY	VECTOR	VECTOR UNITY	CONVERSION FACTOR (kg CO2-eq Vector Unity-1)
Diesel oil	Diesel oil (fabrication and combustion)	Kg	3.7243
Fertilizers	Nitrogen (fabrication)	Kg	12.68
	N_2_ emissions	Kg	6.205 (indirect emissions included)
	Phosphorous o (P_2_O_5_)(fabrication)	Kg	2.2344
	Potassium (K_2_O) (fabrication)	Kg	2.4099
Herbicides, fungicides and insecticides	Herbicides, fungicides and insecticides	Kg	10.3087
Transport of products and raw materials	Truck (transport, freight, lorry, 7.5-16 metric ton)	t·km	0.230849
	Small truck (Lorry3.5-7.5t)	t·km	0.49305
	Van (transport, freight, light commercial vehicle)	t·km	1.9657
	Tractor (transport, tractor and trailer, agricultural (CH))	t·km	0.38842
Infrastructure (Field machinery—tractor)	Iron	Kg	2.54615553
	Rubber	Kg	2.874869847

## Appendix A

**Table A1:** CO_2_ footprint (kg CO_2_-eq kg^−1^ product and kg CO_2_- eq ha^−1^) for each program along the three growing seasons.

Programs	CO_2_ Footprint (kg CO_2_eq kg^−1^ Product)			Average	CO_2_ Footprint (kgCO_2_eq ha^−1^)			Average
	2014–2015	2015–2016	2016–2017		2014–2015	2015–2016	2016–2017	
Program 1 TRAD								
CO_2_ DIESEL OIL field works	0.1	0.0	0.1	0.1	191	266	182	213
CO_2_ FERTILIZERS fabrication	0.6	0.2	0.8	0.5	1742	1167	1990	1633
CO_2_ FERTILIZERS application	0.2	0.1	0.3	0.2	623	987	745	785
CO_2_ AGROCHEMICALS fabrication	0.0	0.0	0.0	0.0	46	47	46	47
CO_2_ transport of product	0.0	0.0	0.0	0.0	4	4	4	4
CO_2_ transport of raw materials	0.0	0.0	0.0	0.0	114	78	126	106
CO_2_ INFRAESTRUCTURE tractor	0.0	0.0	0.0	0.0	4	5	3	4
TOTAL	**0.9**	**0.4**	**1.2**	**0.8**	**2725**	**2553**	**3096**	**2791**
PROGRAM 2 CER								
CO_2_ DIESEL OIL field works	0.1	0.0	0.1	0.1	191	266	180	213
CO_2_ FERTILIZERS fabrication	0.6	0.2	0.7	0.5	1742	1167	1990	1633
CO_2_ FERTILIZERS application	0.2	0.1	0.3	0.2	623	987	745	785
CO_2_ AGROCHEMICALS fabrication	0.0	0.0	0.0	0.0	31	52	67	50
CO_2_ transport of product	0.0	0.0	0.0	0.0	4	4	4	4
CO_2_ transport of raw materials	0.0	0.0	0.0	0.0	114	78	126	106
CO_2_ INFRAESTRUCTURE tractor	0.0	0.0	0.0	0.0	4	5	3	4
TOTAL	**1**	**0.4**	**1.1**	**0.8**	**2710**	**2558**	**3115**	**2794**
PROGRAM 3 CR								
CO_2_ DIESEL OIL field works	1.1	0.1	0.1	0.4	199	273	199	224
CO_2_ FERTILIZERS fabrication	9.5	0.3	0.6	3.5	1742	1167	1990	1633
CO_2_ FERTILIZERS application	3.4	0.2	0.2	1.3	623	987	745	785
CO_2_ AGROCHEMICALS fabrication	0.5	0.0	0.0	0.2	88	73	82	81
CO_2_ transport of product	0.0	0.0	0.0	0.0	4	4	4	4
CO_2_ transport of raw materials	0.6	0.0	0.0	0.2	114	78	126	106
CO_2_ INFRAESTRUCTURE tractor	0.0	0.0	0.0	0.0	4	5	4	4
TOTAL	**15.2**	**0.6**	**0.9**	**5.6**	**2774**	**2586**	**3149**	**2837**
PROGRAM 4 LI								
CO_2_ DIESEL OIL field works	0.2	0.0	0.1	0.1	203	123	195	174
CO_2_ FERTILIZERS fabrication	1.8	0.0	0.5	0.8	1742	0	1990	1244
CO_2_ FERTILIZERS application	0.6	0.0	0.2	0.3	623	0	745	456
CO_2_ AGROCHEMICALS fabrication	0.0	0.0	0.0	0.0	31	57	82	57
CO_2_ transport of product	0.0	0.0	0.0	0.0	4	0	4	3
CO_2_ transport of raw materials	0.1	0.0	0.0	0.0	114	0	126	80
CO_2_ INFRAESTRUCTURE tractor	0.0	0.0	0.0	0.0	4	3	4	3
TOTAL	**2.8**	**0.0**	**0.8**	**1.2**	**2722**	**182**	**3146**	**2016**

TRAD, barley monocrop with post emergence herbicide applications; CER, wheat-barley rotation with pre- and post-emergence herbicide applications; CR, crop rotation (pea-barley-wheat) with chemical and cultural management; LI, low input non-chemical management.
